# Identification of a novel gut microbiota signature associated with colorectal cancer in Thai population

**DOI:** 10.1038/s41598-023-33794-9

**Published:** 2023-04-24

**Authors:** Nutta Iadsee, Natthaya Chuaypen, Teerasit Techawiwattanaboon, Thananya Jinato, Tanisa Patcharatrakul, Songphol Malakorn, Aisawan Petchlorlian, Kearkiat Praditpornsilpa, Kanitha Patarakul

**Affiliations:** 1 Medical Microbiology, Interdisciplinary Program, Graduate School, Chulalongkorn University, Bangkok, Thailand; 2 Department of Biochemistry, Faculty of Medicine, Center of Excellence in Hepatitis and Liver Cancer, Chulalongkorn University, Bangkok, Thailand; 3 Department of Microbiology, Faculty of Medicine, Chulalongkorn University, Bangkok, Thailand; 4 Chula Vaccine Research Center (Chula VRC), Center of Excellence in Vaccine Research and Development, Chulalongkorn University, Bangkok, Thailand; 5 Doctor of Philosophy Program in Medical Sciences, Graduate Affairs, Faculty of Medicine, Chulalongkorn University, Bangkok, Thailand; 6 Division of Gastroenterology, Department of Medicine, King Chulalongkorn Memorial Hospital, The Thai Red Cross Society, Bangkok, Thailand; 7 Faculty of Medicine, Center of Excellence in Neurogastroenterology and Motility, Chulalongkorn University, Bangkok, Thailand; 8 Division of Colorectal Surgery, Department of Surgery, Faculty of Medicine, Chulalongkorn University, Bangkok, Thailand; 9 Division of Geriatric Medicine, Department of Medicine, Faculty of Medicine, Chulalongkorn University, Bangkok, Thailand; 10 Geriatric Excellence Center, King Chulalongkorn Memorial Hospital, The Thai Red Cross Society, Bangkok, Thailand; 11 Division of Nephrology, Department of Medicine, Faculty of Medicine, Chulalongkorn University, Bangkok, Thailand

**Keywords:** Cancer, Computational biology and bioinformatics, Microbiology, Molecular biology, Biomarkers

## Abstract

Colorectal cancer (CRC) is the third most common cancer worldwide. Dysbiosis of human gut microbiota has been linked to sporadic CRC. This study aimed to compare the gut microbiota profiles of 80 Thai volunteers over 50 years of age among 25 CRC patients, 33 patients with adenomatous polyp, and 22 healthy controls. The 16S rRNA sequencing was utilized to characterize the gut microbiome in both mucosal tissue and stool samples. The results revealed that the luminal microbiota incompletely represented the intestinal bacteria at the mucus layer. The mucosal microbiota in beta diversity differed significantly among the three groups. The stepwise increase of *Bacteroides* and *Parabacteroides* according to the adenomas–carcinomas sequence was found. Moreover, linear discriminant analysis effect size showed a higher level of *Erysipelatoclostridium ramosum* (ER), an opportunistic pathogen in the immunocompromised host, in both sample types of CRC patients. These findings indicated that the imbalance of intestinal microorganisms might involve in CRC tumorigenesis. Additionally, absolute quantitation of bacterial burden by quantitative real–time PCR (qPCR) confirmed the increasing ER levels in both sample types of cancer cases. Using ER as a stool–based biomarker for CRC detection by qPCR could predict CRC in stool samples with a specificity of 72.7% and a sensitivity of 64.7%. These results suggested ER might be a potential noninvasive marker for CRC screening development. However, a larger sample size is required to validate this candidate biomarker in diagnosing CRC.

## Introduction

Colorectal cancer (CRC) is the third leading cause of cancer–related deaths worldwide, with an estimated 1.9 million new cases and 935,000 deaths annually^1^. Approximately 60–65% of sporadic CRCs, which occur spontaneously without a family history of CRC or inherited genetic mutation, are the major proportion of CRC cases^2^. Adenomatous polyps or adenomas are precancerous lesions in almost all sporadic CRCs and were found in up to 50% of persons above 50 years of age undergoing colonoscopy^3^. Early CRC screening and removal of benign or precancerous polyps are recommended for effective prevention^4^. Apart from known risk factors such as age 50 or greater, unhealthy dietary habits, ethnicity, and smoking^5^, emerging evidence indicates that dysbiosis of intestinal bacteria is associated with the pathogenesis of sporadic CRC^6^. Numerous bacterial species have been studied, of which representative culturable isolates interplayed with cancer cell lines and induced disease pathogenesis in vivo^7–11^. However, there was no consensus on microbial signatures in CRC patients. The inconsistency might be caused by different technical approaches and geographical locations^12^.

Furthermore, microbiota in the colon mucosa (mucosal microbiota) and the feces (luminal microbiota) are different, although their patterns were shown to be partially correlated^13,14^. Gut microbiome profiles from both stool and colon mucosa of Asian people have been scarcely reported. In this study, we conducted a comparative analysis of the gut microbiome profile using stool and mucosal samples from Thai CRC patients, individuals with adenomas, and healthy volunteers aged 50 and above using 16S rRNA sequencing analysis. We then performed the quantitative PCR to validate the predominant bacterial signature in both sample types from Thai CRC patients.

## Results

### Demographic and clinical data of CRC patients, individuals with adenomas, and healthy volunteers

A total of 80 Thai participants (range; 51–85 years old) consisting of 25 CRC, 33 adenomas (AD), and 22 healthy control (HC) groups were recruited from June 2019 to December 2020. Detailed demographic distribution among the three groups is summarized in Table 1. There were no differences among CRC, AD, and HC groups according to gender, age, BMI, or underlying diseases, i.e., hypertension (HT) and dyslipidemia (DLP), except for type 2 diabetes mellitus (T2DM) (more T2DM in the CRC patients, *p* < 0.05). In the CRC group, most of the tumors were classified in stage T3 (60%). Other clinicopathological data were described in Supplementary Table S1.

**Table 1:** Demographic and clinical data of the participants in this study.

Variable	Group			Total	*p–*value
	HC	AD	CRC		
No. of volunteers	22	33	25	80	
Age (Mean ± SD)	62.2 ± 4.7	66.3 ± 5.3	65.2 ± 8.1	64.8 ± 6.3	0.068
BMI (Mean ± SD)	23.0 ± 3.1	24.0 ± 3.6	22.2 ± 2.8	23.1 ± 3.3	0.055
Gender (n, (%))					
Male	5 (22.7)	15 (45.5)	9 (36)	29 (36.25)	0.229
Female	17 (77.3)	18 (54.5)	16 (64	51 (63.75)	
Type 2 diabetes mellitus, T2DM (n, (%))					
Yes	0 (0)	4 (12.1)	7 (28)	11 (13.75)	0.020*
No	22 (100)	29 (87.9)	18 (72)	69 (86.25)	
Hypertension, HT (n, (%))					
Yes	8 (36.4)	15 (45.5)	10 (40)	33 (41.25)	0.789
No	14 (63.6)	18 (54.5)	15 (60)	47 (58.75)	
Dyslipidemia, DLP (n, (%))					
Yes	11 (50)	17 (51.5)	10 (40)	38 (47.5)	0.660
No	11 (50)	16 (48.5)	15 (60)	42 (52.5)	
Tumor location (n, (%))					
Proximal colon	–	20 (60.6)	4 (16)	24 (41.4)	
Distal colon	–	13 (39.4)	21 (84)	34 (58.6)	
Tumor staging (T) (n, (%))					
T1	–	–	2 (8)	2 (8)	
T2	–	–	5 (20)	5 (20)	
T3	–	–	15 (60)	15 (60)	
T4	–	–	3 (12)	3 (12)	

Statistical significance was determined by the nonparametric Kruskal–Wallis test. Gender and underlying diseases were evaluated with the chi–squared test. Data are shown as mean ± SD. *HC* healthy control; *AD* patients with adenomas; *CRC* patients with colorectal cancer. *p < 0.05.

### Alpha diversity and beta–diversity metrics of the mucosal microbiota

**Figure 1:**
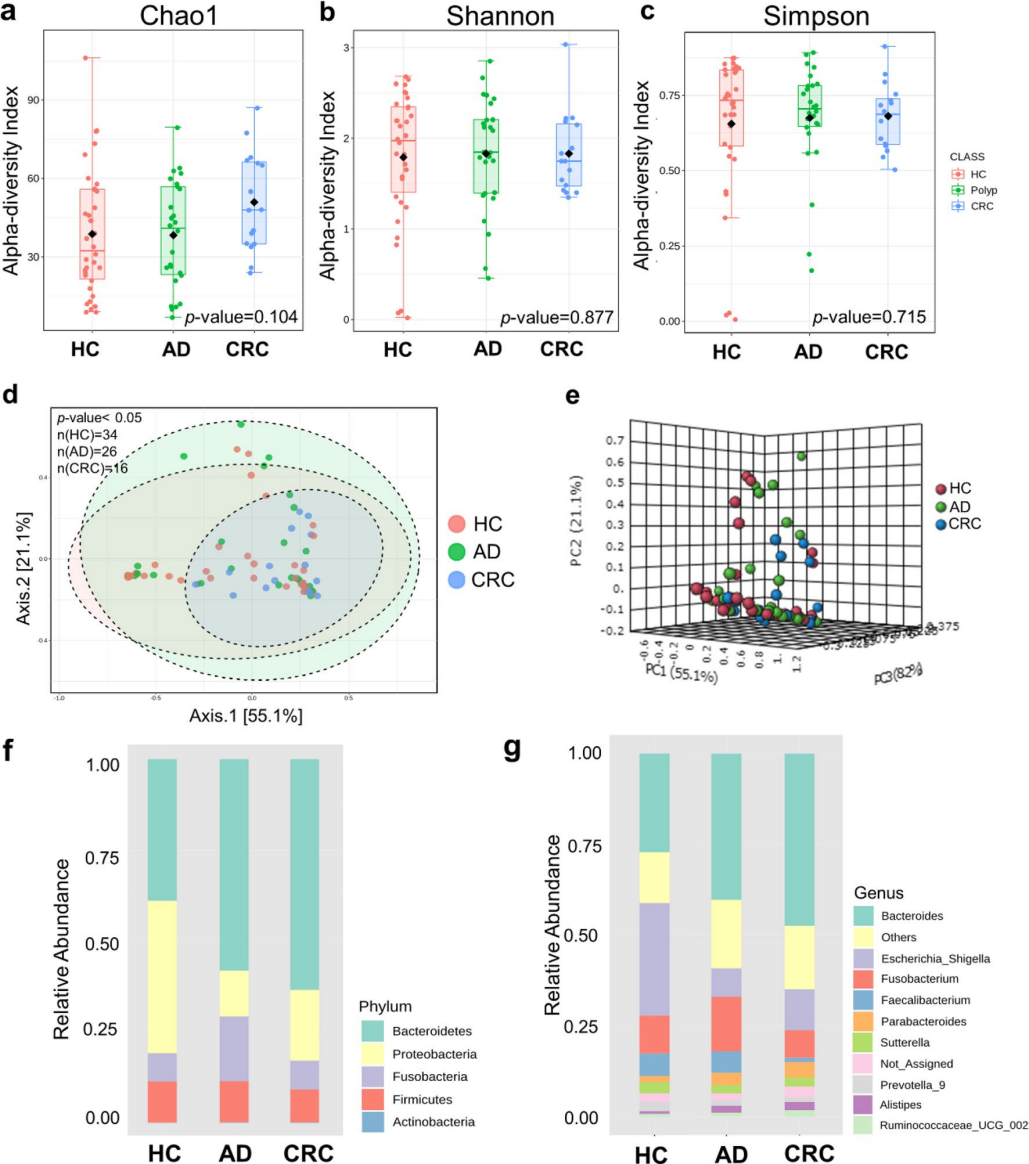
Microbial diversity analyses and microbial abundance profiling in mucosal tissue samples among healthy control (HC), adenoma (AD), and colorectal cancer (CRC) subjects, (**a**) Chao1’s index, (**b**) Shannon’s index, (**c**) Simpson’s index, (**d**) Principal Coordinate Analysis (PCoA) analysis based on Bray–Curtis distance in 2–Dimension, (**e**) PCoA analysis based on Bray–Curtis distance in 3–Dimension,** (f**) Taxonomic composition type at the phylum level, (**g**) Taxonomic composition at the genus level.

There were no statistically significant differences in alpha–diversity indices including Chao1’s index (*p* = 0.104) (Fig. 1a), Shannon’s index (*p* = 0.877) (Fig. 1b), and Simpson’s index (*p* = 0.715) (Fig. 1c) among CRC, AD and HC groups. However, a significant difference in beta diversity was observed in Bray–Curtis’s distance among the three groups (*p* = 0.039) (Fig. 1d and e).

### Altered gut mucosal microbiota in CRC and AD groups

The relative taxonomic pattern of mucosal microbiota from CRC, AD, and HC groups was examined. Overall microbial compositions of the AD and the CRC groups were shifted compared to that of healthy controls. At the phylum level, Bacteroidetes was the most prevalent in the AD and the CRC groups (63% and 58%, respectively), while Proteobacteria predominated in the HC group (42%) (Fig. 1f). At the genus level, a stepwise increase of *Bacteroides* and *Parabacteroides* in the CRC group (55.2% and 3.5%) was observed when compared with the AD (50.4% and 3.4%) and the HC groups (36.0% and 1.2%). On the other hand, *Escherichia*–*Shigella* in the AD (8.8%) and the CRC (16.4%) groups decreased compared with the HC group (35%). Furthermore, there was a decrease in *Faecalibacterium* in the CRC patients (0.7%) compared with the AD (2.4%) and the HC groups (4%) (Fig. 1g).

**Figure 2:**
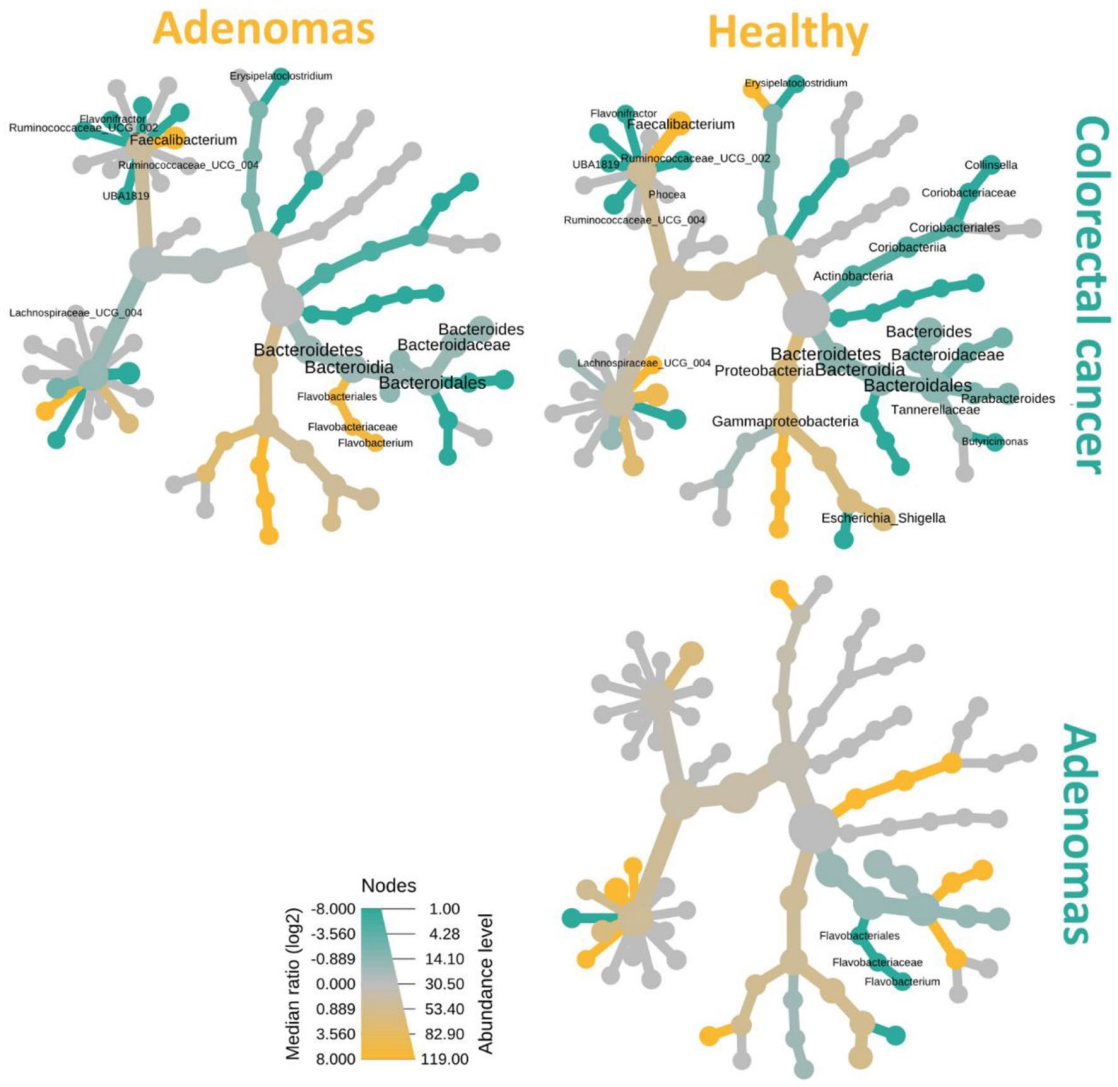
Heat tree analysis illustrating the taxonomic alterations in the mucosal microbiota composition according to adenoma–carcinoma sequence based on the log2 ratio median abundance. Significant alterations of taxa are displayed by name at the corresponding node. The nodes indicate the hierarchical structure of taxa. Three comparisons were conducted: colorectal cancer (CRC) (green) vs healthy control (HC) (yellow); CRC (green) vs adenoma (AD) (yellow); and AD (green) vs HC (yellow). The dominant color corresponds to a higher number of amplicon sequence variants (ASVs). The Log2 ratio is 0 (gray) when the compared groups are not significantly different.

Determining group–wise alteration in the bacterial abundance of AD or CRC at the genus level, the heat trees displayed only significant differences in relative abundance of the individual taxon in which the terminal nodes corresponding to the bacterial genera (Fig. 2). Three comparisons between groups were accomplished as follows: (1) HC versus (vs.) CRC; (2) AD vs CRC (3) HC vs AD. The highest difference in bacterial composition was observed between CRC patients and healthy controls. Compared to HC, the bacterial diminution in CRC was detected in the genus *Faecalibacterium*, *Escherichia–Shigella*, and within the *Lachnospiraceae* family. In the opposite direction, bacteria within the phylum Bacteroidetes, the genus *Bacteroides* (*p* = 0.012), *Parabacteroides* (*p* = 0.004), and *Butyricimonas* (*p* = 0.042) increased in the HC group compared to the CRC group. Moreover, genus *Collinsella* (*p* = 0.026), *Erysipelatoclostridium* (*p* = 0.0001), *Ruminococacease* (*p* = 0.002), *Flavonifractor* (*p* = 0.009), and *Phocea* (*p* = 0.029) were significantly predominated in the CRC patients. As for bacterial comparisons between AD subjects and others, only alteration of genus *Flavobacterium* belonging to the phylum Bacteroidetes was significantly over–represented in the AD group compared with the HC group. Interestingly, a significant rise in this genus was found in the AD group compared with the CRC group.

**Figure 3:**
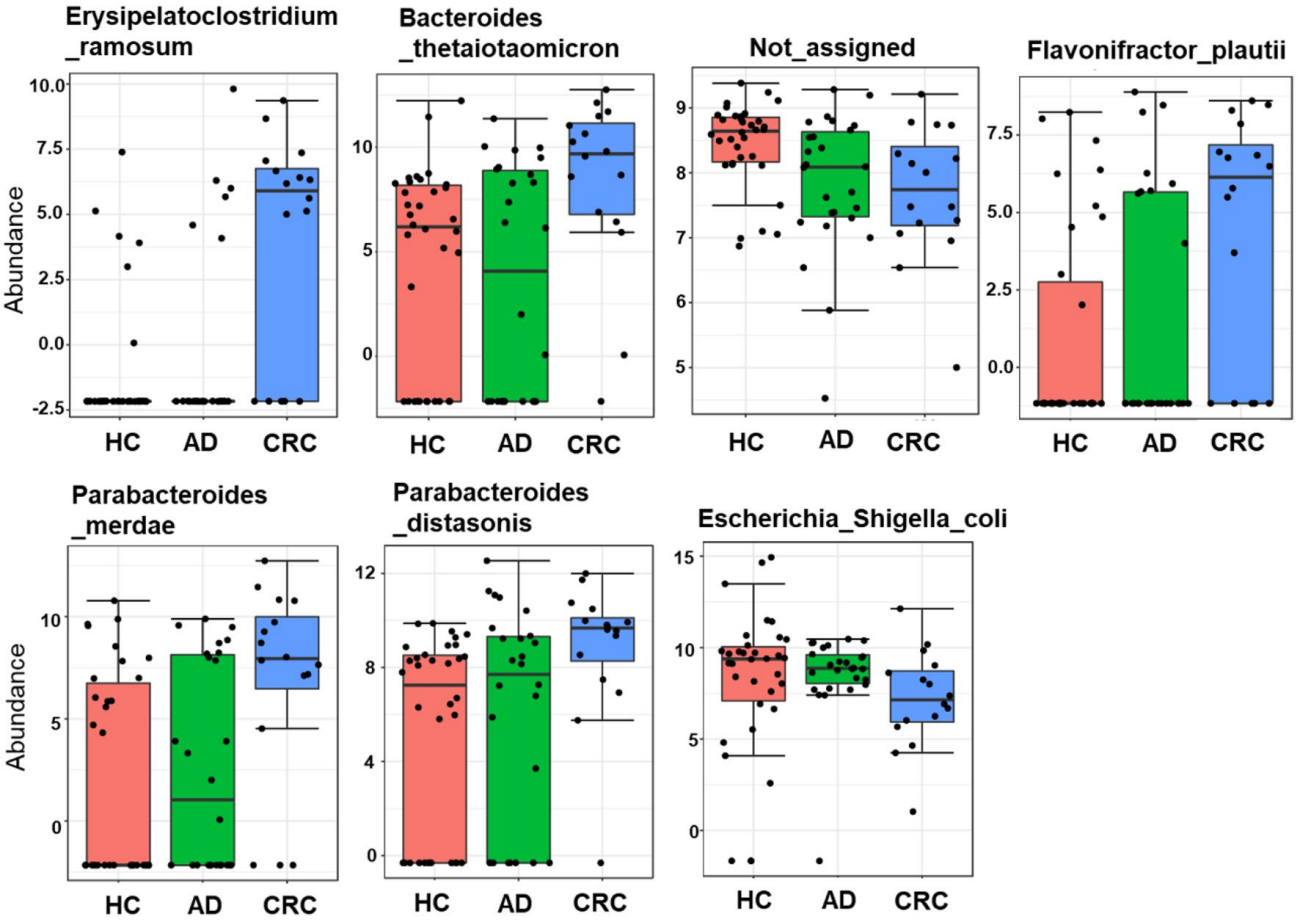
The significant difference in bacterial abundance of the individual bacterium at the species level in the mucosal tissue samples among healthy control (HC), adenoma (AD), and colorectal cancer (CRC) subjects. Data are shown as log–transformed count and *p* < 0.05.

For exploring a differential abundance of gut microbiota at the species level, the univariate analysis in MicrobiomeAnalyst was carried out with default parameters (*p* < 0.05). The analysis identified seven bacterial species that were significantly abundant among the three groups (Fig. 3). Five species representing *Erysipelatoclostridium ramosum* (*p* < 0.001), *Bacteroides thetaiotaomicron* (*p* = 0.002), *Flavonifractor plautii* (*p* = 0.021), *Parabacteriodes merdae* (*p* = 0.012), and *Parabacteriodes distasonis (p* = 0.020) were more abundant in the CRC group. While *Escherichia coli–Shigella* sp. (*p* = 0.019) and one of the unassigned taxa (*p* = 0.013) were significantly lower in CRC patients.

### Luminal microbiota partially reflected mucosal microbiota

**Figure 4:**
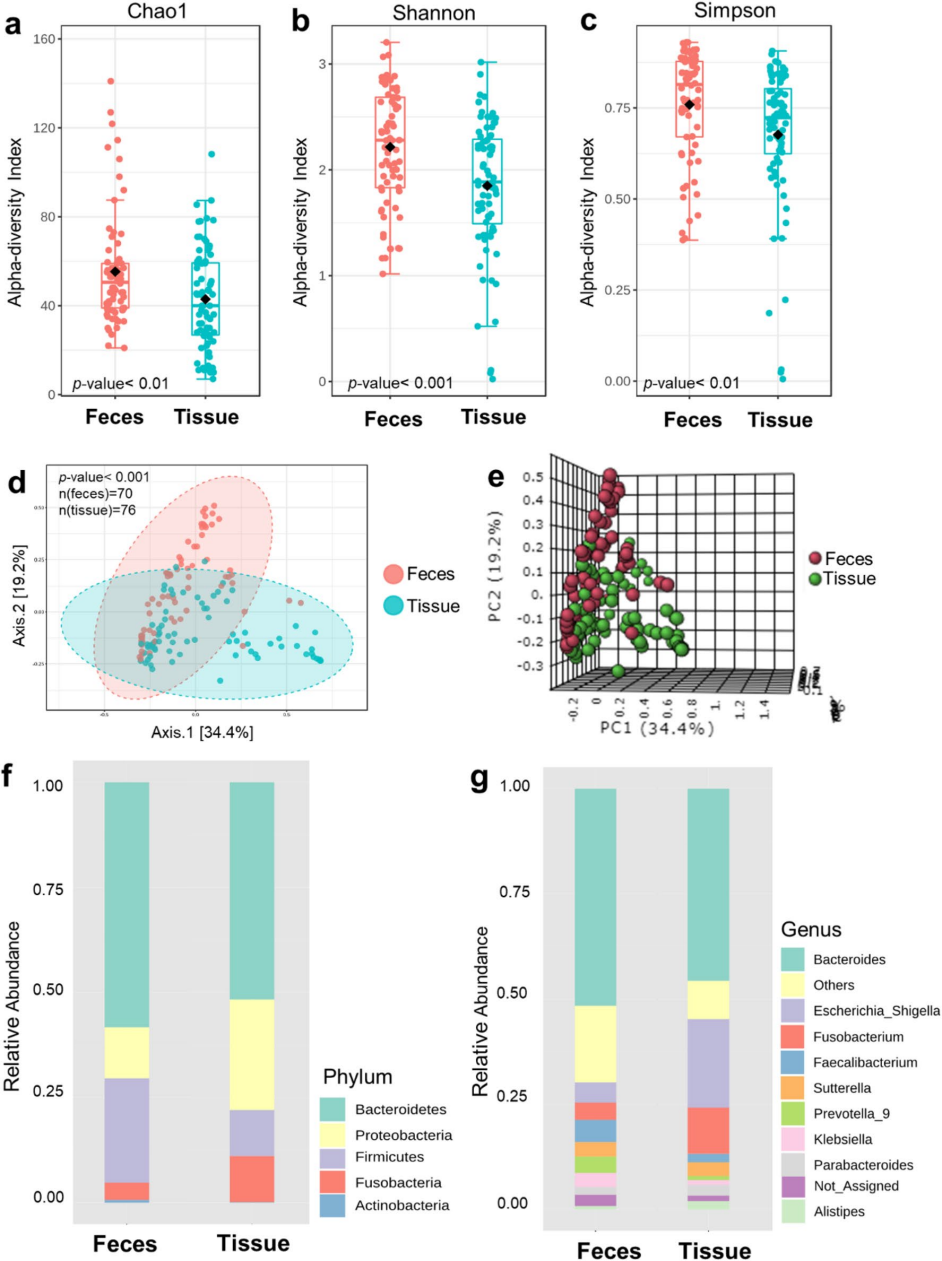
Microbial diversity analyses and microbial abundance profiling between feces and mucosal tissue samples, (**a**) Chao1’s index, (**b**) Shannon’s index, (**c**) Simpson’s index, (**d**) Principal Coordinate Analysis (PCoA) analysis based on Bray–Curtis distance in 2–Dimension, (**e**) PCoA analysis based on Bray–Curtis distance in 3–Dimension,** (f**) Taxonomic composition type at the phylum level, (**g**) Taxonomic composition at the genus level.

Early detection of CRC is essential to reduce the mortality rate. Fecal biomarkers are more practical than utilizing tissue samples for CRC screening. We analyzed whether luminal microbiota could represent a proxy of mucosal microbiota in CRC. Luminal and mucosal microbiota of paired samples from 47 participants were significantly different between sample types in terms of alpha diversity and beta diversity (Supplementary Fig. S1). The distinction of microbial diversity was detected when all available samples (paired and unpaired samples) were included in the analysis (Fig. 4a–e). Bacterial comparison between sample types at the phylum level showed the luminal microbiota harbored more Bacteroidetes, Firmicutes, and Actinobacteria, whereas Proteobacteria and Fusobacterium over–represented in the mucosal microbiota (Fig. 4f). At the genus level, *Escherichia–Shigella*, and *Fusobacterium* were found more abundance in the mucosal microbiota than the luminal microbiota (Fig. 4g). However, the luminal microbiota among CRC patients, individuals with AD, and HC subjects showed no difference in overall microbiota (alpha and beta diversity) (Supplementary Fig. S2a–e). Nonetheless, the univariate analysis of fecal microbiota found significant differences in the abundance of three bacterial species among three groups (*E. ramosum* (*p* < 0.001), *B. vulgatus* (*p* = 0.020), and *Eggerthella lenta* (*p* = 0.024)) (Supplementary Fig. S2f). Thus, even though luminal and mucosal microbiota were significantly different, and luminal microbiota only incompletely reflected the intestinal microorganisms at the host mucus layer, the microbial imbalance associated with cancer was still apparent.

### Identification of a novel microbial signature for CRC screening

**Figure 5:**
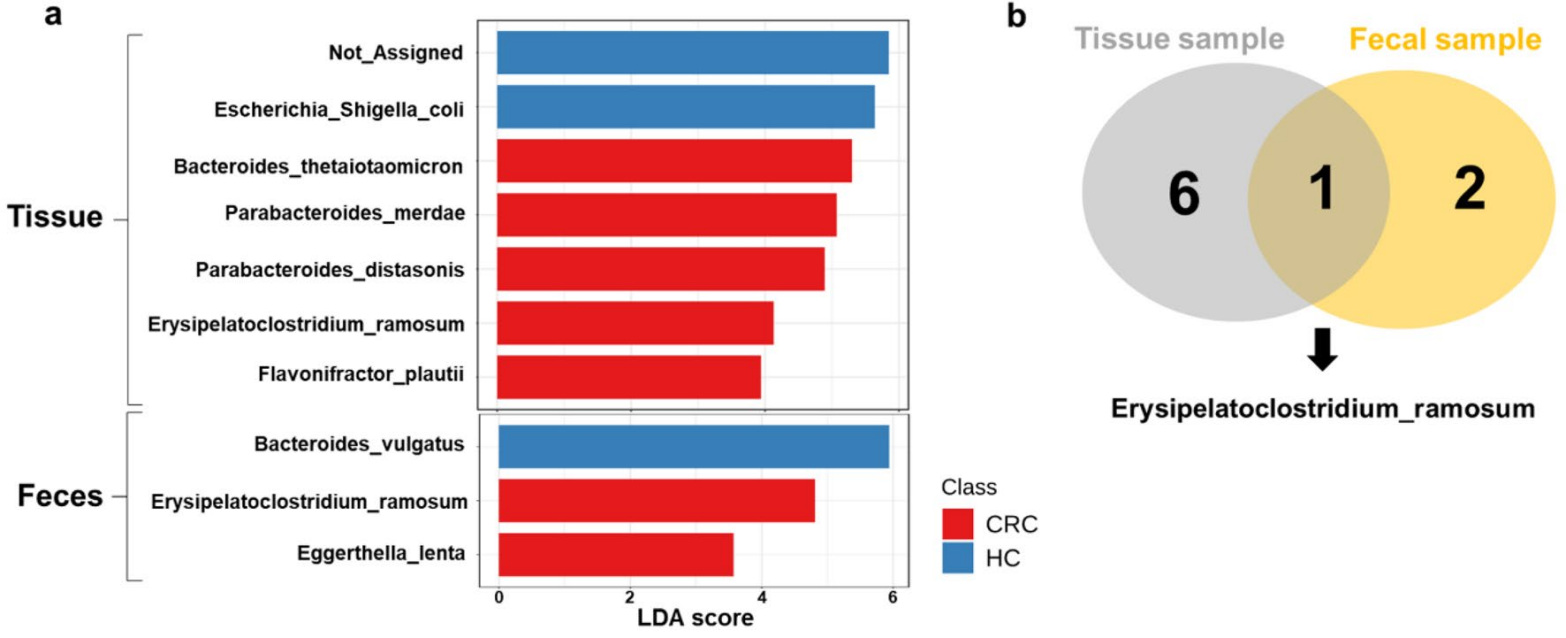
Linear discriminant analysis (LDA) effect size (LEfSe) analysis of mucosa (tissue) and lumen (feces)–associated microbiota among healthy control (HC), adenoma (AD), and colorectal cancer (CRC) groups, (**a**) Histogram of the LDA scores for significantly abundant species, (**b**) Venn diagram represented the number of unique and overlapping significantly abundant species.

To further confirm the taxonomic differences among CRC, AD, and HC groups, biomarker discovery using LEfSe analysis was performed with an LDA score ≥ 3.0 for both sample types. As shown in Fig. 5a, the mucosal microbiota of CRC was over–represented by *B. thetaiotaomicron*, *P. merdae*, *P. distasonis*, *E. ramosum*, and *F. plautii*. In addition, unspecified bacteria and *Escherichia coli–Shigella* sp. predominated in HC tissue. In fecal samples, *E. ramosum* and *E. lenta* were enriched in the CRC group, while *B. vulgatus* was predominant in the HC group. Additionally, only *E. ramosum* was explicitly detected in the CRC patients in fecal and tissue samples (Fig. 5b). However, this study did not find putative biomarkers that could differentiate AD patients from the HC group.

### Bacterial quantification in the clinical samples by absolute qPCR

**Figure 6:**
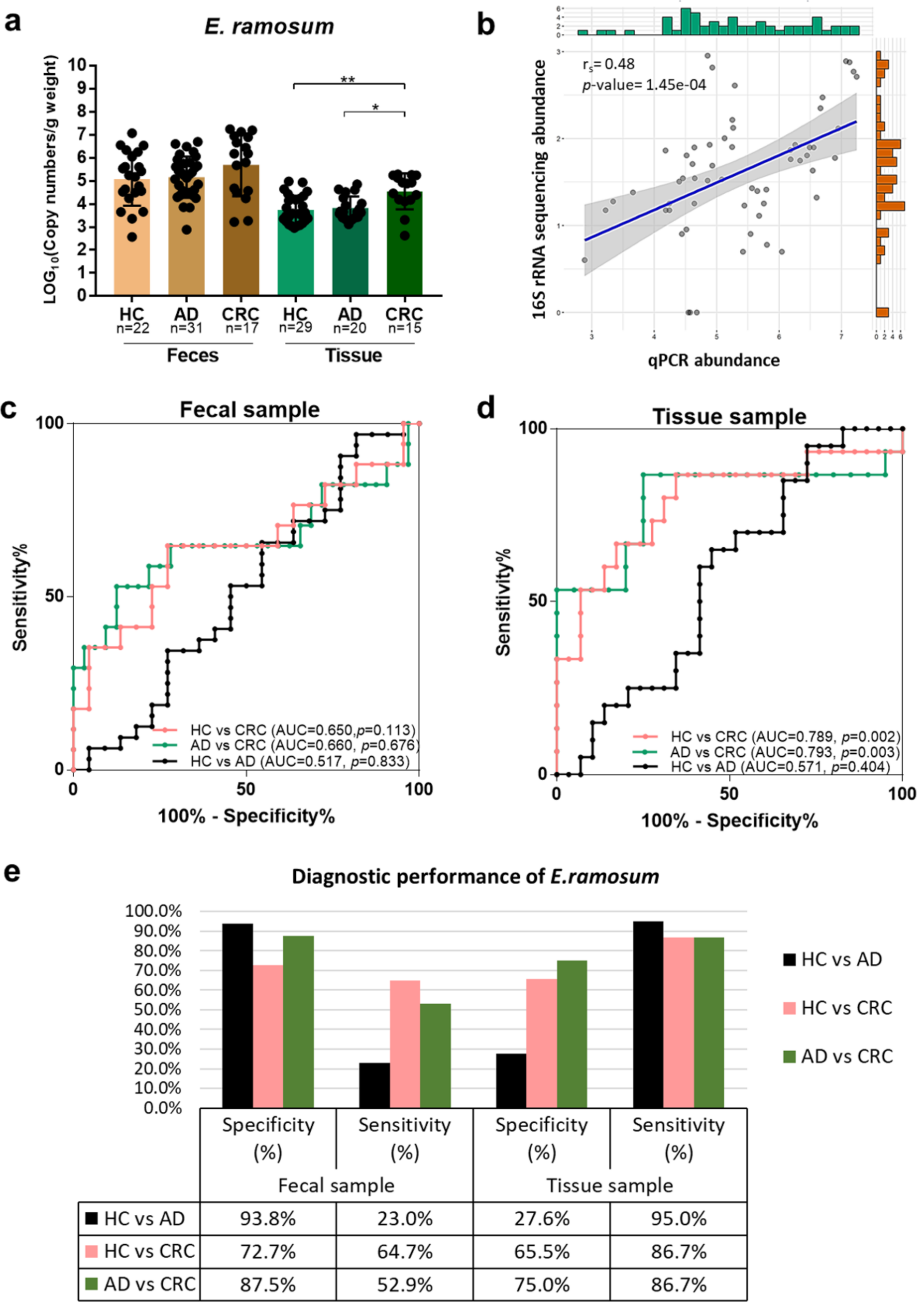
Absolute quantification and diagnostic performance of CRC–associated *Erysipelatoclostridium ramosum* in the clinical samples, (**a**) absolute quantity of *E. ramosum* of both fecal and tissue samples. The scatter plot is expressed as log10 copy number per gram weight, and the data are displayed as mean ± SD. Each dot represents one sample. (**b**) Spearman’s rank correlation coefficient between 16S rRNA gene sequencing and qPCR assay abundance for *E. ramosum*. Data was shown in log10 of reads and copy number per gram weight, respectively. (**c**) ROC curve for colorectal cancer (CRC) and adenoma (AD) detection in fecal samples. (**d**) ROC curve for CRC and AD detection in tissue samples. (**e**) Diagnostic performance of *E. ramosum* as CRC biomarker. Abbreviations: *p < 0.05; **p < 0.01.

To validate the result of *E. ramosum* (ER) in both stool and tissue samples of CRC patients, the quantification of ER in another aliquot of DNA from the identical samples used in this study was conducted by absolute qPCR. Based on the designed primers targeting the 16S rRNA gene of ER, the nucleotide sequence of the constructed plasmid used as a positive control revealed 93.1% similarity to that of *E. ramosum* strain D40–2 (accession number MT275475.1). We found that the copy number per gram weight (CN/g) of ER was significantly enriched in tissue samples of CRC patients compared with the AD (*p* = 0.027) and healthy donors (*p* = 0.002) (Fig. 6a). Nevertheless, there was no significant difference in the abundance of fecal ER among the three groups (Fig. 6a). Bivariate correlation analysis showed that the absolute quantity of ER measured by qPCR assay was significantly correlated with the 16S rRNA sequencing abundance (Spearman’s r = 0.48, p < 0.001) (Fig. 6b). Binary logistic regression models were generated using the ER quantity to differentiate according to three pair–wise analyses: (1) HC vs CRC; (2) AD vs CRC; (3) HC vs AD. Of these predictive models in the stool (Fig. 6c) and tissue samples (Fig. 6d), ROC analysis showed that mucosal ER provided greater discrimination in CRC detection than fecal ER. Mucosal ER could distinguish patients with CRC from HC, and CRC from individuals with AD at similar performance, giving an area under the ROC curve (AUC) of 0.789 (sensitivity (sn) = 86.7%, specificity (sp) = 65.5%), and 0.793 (sn = 86.7%, sp = 75.0%), respectively (Fig. 6d and e). Moreover, at the best cut–off value (4.44E + 05 CN/g), the fecal ER could discriminate cancer patients from HC with a sensitivity of 64.7%, and specificity of 72.7% (Fig. 6e). However, the bacterial markers of ER in both sample types indicated poorer performance in AD detection (HC vs AD) than those in cancer detection (HC vs CRC, and AD vs CRC) (Fig. 6c–e).

## Discussion

Accumulating evidence has indicated that alteration of intestinal microbiota composition possibly affects the initiation and progression of CRC^15,16^. Although adherent bacteria might be more inclined to interact with host cells than transient bacteria that are shed daily in the stool, there were a limited number of studies on gut microbiome analysis using colon tissue samples of CRC patients^17–19^. Matched non–tumor tissues from CRC patients were usually used for comparison^20,21^. In addition, worldwide studies, especially data from the Asia region, are rarely conducted in stool and mucosal samples to comprehensively analyze luminal and mucosal microbiota in CRC^13,22,23^. In this study, the 16S rRNA gene sequencing tool was utilized to compare the bacterial composition in stool and mucosal tissues among three groups of Thai participants aged 50 years or above, consisting of healthy volunteers, patients with AD as CRC precancerous lesions, and patients with CRC. The structural segregation of gut microbiota between mucosal tissues and fecal samples showed significant differences in bacterial richness, diversity, and overall microbial profile. However, fecal dysbiosis related to disease status was still observed (Supplementary Fig. S3) and partially overlapped with mucosal microbiota. These findings confirm the previous studies suggesting luminal microbiota incompletely represented the microbiota on the mucus layer^13,22^.

Tjalsma and his team proposed the bacterial driver–passenger model for CRC development^24^. Certain bacteria in the colon can initiate multistep colorectal carcinogenesis consisting of induced inflammation, increased cell proliferation, and/or genotoxin production. After epithelial DNA damage, colorectal tumorigenesis might be associated with alteration of the gut microenvironment that facilitates the outnumber of colonic commensals with either tumor–promoting or tumor–suppressive features considered as passenger bacteria. Furthermore, bacterial drivers may be replaced by passenger bacteria that could take the growth benefit of the cancerous microenvironment. Concerning the mucosal microbiota in this study, *Flavobacterium*, an opportunistic pathogen in immunocompromised patients^25^, was significantly over–represented in the mucosal tissues of the AD group but not in CRC patients. This observation is consistent with a previous report on patients with intestinal metaplasia^26^. This bacterium might be associated with the early or precancerous stage of CRC and act as driver bacteria but is subsequently outcompeted by passenger bacteria in CRC patients.

As for passenger bacteria, the interesting and consistent observation was the significant enrichment of *Flavonifractor plautii* and *Parabacteroides distasonis* on the mucosal tissue in Thai CRC patients, similar to the results of an Indian CRC study^27^. These bacterial species converted beneficial dietary flavonoids, found in a plant–based diet, e.g., green tea, wine, and cocoa, into the human intestine^28^. Although flavonoids are primarily composed of polyphenolic compounds with a wide spectrum of pharmacological properties, including their influential role in anti–cancer activity^29^, spacious degradation of flavonoids by intestinal microorganisms may lead to lower total bioavailability and lower opportunity for intact flavonoids to be absorbed in the epithelial cells^30^. Therefore, the elevated abundance of these bacteria may be related to the higher activity of flavonoid degradation that reduces the anti–carcinogenic effects and bioavailability of flavonoids in CRC patients. Meanwhile, the protective role of *P. distasonis* in tumor development and their property in the maintenance of gut barrier has been proposed in tumor–bearing mice^31^. Nevertheless, in this study, we cannot conclude the definite functional pathway co–involved with gut microbiota in Thai CRC subjects. Thus, further studies are required to investigate whether these bacteria play the potential role of passenger bacteria with tumor–suppressive or tumor–promoting features.

On the other hand, the significant overrepresentation of the genus *Collinsella* in individuals with CRC was observed and agreed with previous studies^32,33^. *Collinsella* was shown to have a role in elevating gut barrier permeability and inducing the production of proinflammatory IL–17 in patients with rheumatoid arthritis^34^. Another remarkable observation of mucosal microbiota, *Erysipelatoclostridium ramosum* was less abundant in the HC and AD groups while it was significantly elevated in the cancer tissues. This bacterium could be an opportunistic pathogen in immunocompromised hosts^35^, but no study has evaluated its function in CRC. Some strains of *E. ramosum* were shown to secrete human immunoglobin A proteases^36^. The IgA proteases can cleave anti–microbial IgA translocating across the colonic mucosa resulting in increased host susceptibility. These results suggest that *E. ramosum* might serve as one of the passenger bacteria with tumor–promoting roles that preferentially colonize in the tumor microenvironment.

*Escherichia coli–Shigella* sp. were more abundant in the HC tissues and generally referred to as normal flora with potential pathogenic aspects in the large intestine^37^. The abundance of the bacteria in a healthy population was previously reported^38^. However, certain *E. coli* strains can produce genotoxin^39^ and are usually considered as causing agents in the CRC–associated driver–passenger model^24^. The discrepancy between our study and other studies might be explained by 1) the ethnic difference in the susceptibility to colonization by *Escherichia coli–Shigella* sp., 2) the difference in virulence mechanisms and functional roles among strains, i.e., the genera could not be differentiated between normal flora or pathogen in our study, and 3) the possibility of currently healthy volunteers to be a high–risk group for CRC.

Although mucosal bacteria directly interact with colon tissue, using tissue samples in CRC screening is invasive. Fecal samples are more practical for the detection of CRC–related biomarkers. Interestingly, based on LEfSe analysis, *E. ramosum* was solely found in significant abundance in both stool and tissue samples of CRC patients. In our validation via qPCR, the absolute quantification of mucosal *E. ramosum* confirmed the results from 16S rRNA sequencing. In addition, the increasing tendency of fecal *E. ramosum* was observed in CRC patients. This bacterium could be a putative biomarker candidate in CRC screening, providing a potential specificity and sensitivity. Furthermore, previous studies investigated the utilization of fecal microbial biomarkers, such as *Fusobacterium nucleatum* and/or a panel of microbes, has been shown to improve the detection of CRC in independent clinical studies^40,41^ and systematic reviews^42–44^. Additionally, quantitation of microbial biomarkers could improve the accuracy of the fecal immunochemical test (FIT), a current non–invasive screening test, for the prediction of advanced neoplasia and CRC^40,45,46^. Therefore, the bacterial markers should potentially facilitate the early identification of individuals at risk and in need of close surveillance. However, the predominant abundance of *E. ramosum* associated with CRC in our study has not been widely reported in studies from other countries^6,12^. Dissimilar microbial profiles between studies could be the result of distinctions in external factors, including living environments, diet, and lifestyle, or variations in technical characteristics^12^. Multicentric clinical studies using the same workflow and methods are crucial to validate promising candidate biomarkers in a larger group of participants in different geographic locations. The results should reveal common and unique microbial biomarkers for the early detection of CRC in general and specific populations, respectively.

This study has some limitations that might explain different results compared with other studies. Firstly, as for disparate protocols, the selection of universal 16S rRNA gene primers is one of the factors that can cause different results of gut microbiota profiling between studies^12,47^. Most microbiome studies used the primers targeting V3^38,48^, V4^23,49–51^, or V3/V4^13,52,53^ of hypervariable regions of the 16S rRNA gene. However, our preliminary study using all these primers showed low detection sensitivity in the tissue samples (data not shown). Therefore, we performed the 16S rRNA gene sequencing using V1/V2–targeted primers, consistent with previous studies^18,54,55^. Secondly, regarding confounders, the CRC group had significantly more patients with type 2 diabetes mellitus (T2DM) than the HC group. T2DM is associated with an increased risk of CRC^56^. Therefore, it could bias the CRC–associated microbiota result. However, if T2DM patients were excluded from all groups, the impact of mucosal *E. ramosum* still significantly increased in CRC patients compared to HC subjects (Supplementary Fig. S4). Because CRC is a multifactorial disease, this study still included the results from cases with T2DM to perform the microbiome analysis. Thirdly, we designed specific primers to amplify the 16S rRNA gene of *E. ramosum* for qPCR. Nevertheless, the primers should be further verified for specificity and sensitivity before used to quantify *E. ramosum* in clinical application.

In conclusion, this study identified the dysbiosis signature of gut microbiota according to the adenoma–adenocarcinoma sequence for the Thai population. Apart from the host factors, our results implied that the dysbiosis of colonic microorganisms accompanied by driver and passenger bacteria might be involved in CRC tumorigenesis. The microbiome analysis uncovered the putative biomarkers for CRC from mucosal and luminal microbiota. However, a larger sample size is required to further validate the candidate biomarker. Furthermore, the functional profiles of gut microbiota that interact with human hosts or studies in animal models are needed to provide new insights into their roles in CRC carcinogenesis.

## Materials and methods

### Volunteer recruitment, including inclusion and exclusion criteria

A total of 80 Thai volunteers consisting of 25 patients with CRC scheduled for colorectal surgery, 33 subjects with adenomatous polyp, and 22 healthy volunteers who underwent screening colonoscopy for CRC were recruited between June 2019 and December 2020 from the King Chulalongkorn Memorial Hospital (Bangkok, Thailand). All participants were selected according to the following inclusion criteria: Thai nationality, age above 50, and written informed consent. Exclusion criteria were as follows: (1) antibiotics usage within 3 months before sampling; (2) probiotics consumption in any forms within 1 week before enrollment; (3) history of inflammatory bowel disease (IBD); (4) active bowel infection within one month before participation; (5) current immunosuppressive drug usage; (6) previously intake of chemotherapy or radiotherapy; and (7) history of colonoscopy within one month before sample collection. This study was conducted with the approval of the Institutional Review Board (IRB) of the Faculty of Medicine, Chulalongkorn University, Bangkok, Thailand (IRB approval number 182/62) according to the international guidelines for human research protection as the Helsinki Declaration, the Belmont Report, CIOMS Guidelines and International Conference on Harmonization in Good Clinical Practice guidelines. Written informed consent was obtained from each participant.

### Sample collection

All participants were requested to self–collect their feces using a provided stool collection kit before polyethylene glycol (PEG) intake as a bowel preparation step. Fecal samples were immediately collected and preserved in 3 mL DNA/RNA protection reagent (New England Biolabs), transported on an icepack to the laboratory within 12 h, and stored at –80 °C before use. During surgery or colonoscopy, one tissue or biopsy from the peri–lesion site (2–3 mm from the lesion) was collected and preserved in 0.5 mL DNA/RNA protection reagent. For the HC group, randomly one biopsy from the proximal colon (PC) and another biopsy from the distal colon (DC) were collected and preserved in a 0.5 mL DNA/RNA protection reagent and stored at –80 °C before use. In the total of 146 samples, 16 mucosa tissues and 17 fecal samples were obtained from the CRC group. For 7 CRC patients, both stool and tissue samples were obtained. Twenty–six mucosa tissues and 31 fecal samples were obtained from the AD group, and 25 individuals with AD provided paired stool and tissue samples. For healthy volunteers, 34 tissue samples (17 PC and 17 DC) and 22 fecal samples were obtained, and corresponding stool and tissue samples were obtained from 17 healthy subjects.

### DNA extraction

The genomic DNA of stool and tissue samples were extracted using the QIAamp PowerFecal Pro DNA kit (Qiagen) and the QIAamp Fast DNA Tissue Kit (Qiagen), respectively, following the manufacturer’s instructions. The concentration and purity of DNA were determined by NanoDrop2000 spectrophotometer (Thermo Fisher Scientific), and the integrity of DNA was checked by 1.5% (w/v) agarose gel electrophoresis. All DNA samples were stored at –20 °C until further processing.

### 16S rRNA gene sequencing and bioinformatics analysis

Paired–end sequencing was conducted using the Illumina MiSeq 250 bp platform (Illumina) at Génome Québec Innovation Centre (Montréal). The V1–V2 hypervariable regions of the 16S rRNA gene were targeted using the forward primers: 27bF (5′– AGRGTTTGATCMTGGCTCAG–3′) and the reverse primers: 338R (5′–TGCTGCCTCCCGTAGGAGT–3′). The raw 16S rRNA amplicon sequences were cleaned with primer and adapter trimming, then the chimeric sequences were removed using Cutadapt^57^. Briefly, these clean data were clustered as amplicon sequence variants (ASVs) using DADA2^58^. Then taxonomy was annotated with the SILVA v.138.1 16S rRNA reference gene database^59^.

### Microbiome data analysis

The microbiota diversity and abundance were analyzed using the MicrobiomeAnalyst web–based platform (https://www.microbiomeanalyst.ca/)^60^. Gene abundance data were analyzed by Marker Data Profiling (MDP). Data were filtered to remove features with less count of 4 and less than 20% of prevalence, as a minimum, and a low variance filter of 20%, based on the interquartile range. Alpha–diversity profiling was calculated based on the total number of ASV analyzed using the nonparametric tests. Moreover, the beta–diversity was calculated using Bray-Curtis distance and permutational multivariate analysis of variance (PERMANOVA) and visualized by principal coordinate analysis (PCoA) plot. Heat tree analysis was generated for pairwise comparisons of microbial communities. Reingold–Tilfold graph layout and Log2 fold change of relative abundance were displayed. The different taxonomy abundance among groups was also identified with classical univariate statistical comparison. Values were considered statistically significant when the *p* < 0.05. The robust biomarkers of CRC were identified using the linear discriminant analysis (LDA) effect size (LEfSe) approach^61^ with an LDA score ≥ 3.0 and a *p*–value of 0.05.

### Quantification of *Erysipelatoclostridium ramosum* in the clinical samples by quantitative PCR

#### DNA standard curve

In this study, total fecal DNA was used as a PCR template to amplify the 16S rRNA gene of *E. ramosum* used for the positive control plasmid. The specific primers were designed and checked in silico specificity via Primer–BLAST^62^. The nucleotide sequences of the primers targeted were as follows: ERAM2–F: 5′–AGGATGGACTTATGGCGCATT–3′, ERAM2–R: 5′–TACCGTCACTCGGCTACCAT–3′. Based on T/A cloning, the gene fragment amplified using Taq DNA polymerase (Thermo Fisher Scientific) were cloned into the pGEM–T easy vector (Promega, WI, USA) according to the manufacturer’s protocol. The resulting recombinant plasmids were transformed into competent *Escherichia coli* DH5α (Novagen) by heat shock procedure. The DH5α cells carrying the recombinant vector were selected on Luria Bertani (LB) medium with ampicillin (100 µg/ml). The transformant colonies were picked up to check the inserted plasmid via colony PCR using specific primers. The nucleotide sequences of the constructed plasmid were confirmed by DNA sequencing using M13 primers (M13F: 5′–GTAAAACGACGGCCAGT–3′ and M13R: 5′– GCGGATAACAATTTCACACAGG–3′) (Macrogen Inc.). The plasmid was extracted from the colonies using HiYield™ Plasmid Mini Kit (RBC Bioscience). The plasmid copy number was calculated based on the length of the PCR product and the DNA concentration using the following formula: gene copy number = (amount × 6.022 × 10^23^)/(length × 10^9^ × 650). The positive control plasmid was tenfold serially diluted from 2 × 10^7^ to 2 × 10^2^ copies/µl to be used as a standard curve to quantitate the copy number of *E. ramosum* per gram of each sample.

#### Absolute quantitative real–time PCR (absolute qPCR)

The absolute qPCR was performed using QuantStudio6 Flex Real–Time PCR systems (Applied Biosystem) and the Luna Universal qPCR Master Mix (New England Biolabs). The qPCR condition consisted of an initial denaturation at 95 °C for 5 min; 40 cycles of denaturation at 94 °C for 60 s, annealing at 60 °C for 30 s, and extension at 72 °C for 30 s; and a final extension cycle at 72 °C for 8 min. The samples, standard curve, and negative control were all simultaneously assayed in duplicate. The specificity of the PCR product was conducted by the melting curve analysis. The cycle threshold (Ct) of each sample was compared with the Ct of the standard curve to calculate the bacterial quantity. The data were normalized to the total weight of extracted samples and represented as a copy number of bacterium per gram weight.

### Statistical analysis

Statistical analysis was conducted using IBM SPSS Statistics version 22 (SPSS Inc.), GraphPad Prism 8.0 (GraphPad Software Inc.), and R software version 4.1.3. The Chi–square test was used to compare categorical variables. The nonparametric Kruskal–Wallis test with Dunn’s post hoc was used to compare the differences in continuous variables among three clinical groups (i.e., HC, AD, and CRC groups). Spearman’s rank correlation coefficient was used to determine the associations between continuous variables. The independent variables related to CRC or AD diagnosis were estimated using the binary logistic regression model. The area under the receiver operating characteristic (ROC) curve (AUC) was used to evaluate the diagnostic value of potential bacterial markers in discriminating among CRC patients, AD patients, and the HC group. Youden’s index (J = Sensitivity + Specificity–1) was used to identify the best cut–off value that maximizes sensitivity and specificity in disease detection. A *p* < 0.05 was considered statistically significant.

## Supplementary Information

Supplementary Information.

## Data Availability

The sequence data are available in NCBI Sequence Read Archive (SRA) database with the following BioProject ID: PRJNA898111 (https://www.ncbi.nlm.nih.gov/sra/PRJNA898111) and (https://dataview.ncbi.nlm.nih.gov/object/PRJNA898111?reviewer=gv3202bucf9racuatd86do5li5, read–only format).
