# Supplementary material for: Identification of a novel gut microbiota signature associated with colorectal cancer in Thai population
Source: Sci Rep. 2023 Apr 24;13:6702. doi: 10.1038/s41598-023-33794-9 (PMC10126090; doi:10.1038/s41598-023-33794-9)
Supplement: Supplementary file 1 — Supplementary Information. [file 41598_2023_33794_MOESM1_ESM.pdf]

Identification of a Novel Gut Microbiota Signature Associated with Colorectal Cancer in Thai population

Supplementary Information

Supplementary Table S1. Clinicopathological data of the participants in this study.

Variable		Group			Total
		HC	AD	CRC	
**No. of volunteers**		22	33	25	80
**TNM classification**					
**Tumor stage (T)** (n, (%))					
	T1	-	-	2 (8)	
	T2	-	-	5 (20)	
	T3	-	-	15 (60)	
	T4	-	-	3 (12)	
**Node stage (N)** (n, (%))					
	N0	-	-	15 (60)	
	N1	-	-	6 (24)	
	N2	-	-	4 (16)	
**Metastasis stage (M)** (n, (%))					
	Mx	-	-	10 (40)	
	M0	-	-	14 (56)	
	M1	-	-	1 (4)	
**Pathological result** (n, (%))					
I.	Tubular adenoma	-	22 (67)	-	
II.	Tubulovillous adenoma	-	2 (6)	-	
III.	Sessile serrated adenoma	-	1 (3)	-	
IV.	Traditional serrated adenoma	-	1 (3)	-	
V.	Tubular adenoma and tubulovillous adenoma	-	1 (3)	-	
VI.	Tubulovillous and sessile serrated adenoma	-	1 (3)	-	
VII.	Tubular adenoma and hyperplastic polyp	-	3 (9)	-	
VIII.	Tubular adenoma, hyperplastic polyp, and inflammatory polyp	-	2 (6)	-	
IX.	**Adenocarcinoma**	-	-	25 (100)	
**Tumor differentiation** (n, (%))					
	Well	-	-	6 (24)	
	Moderate	-	-	15 (60)	
	Poor	-	-	0 (0)	
	No report	-	-	4 (16)	

Abbreviation: HC – healthy control, AD – patients with adenomas, CRC – patients with colorectal cancer, Mx – metastasis cannot be determined

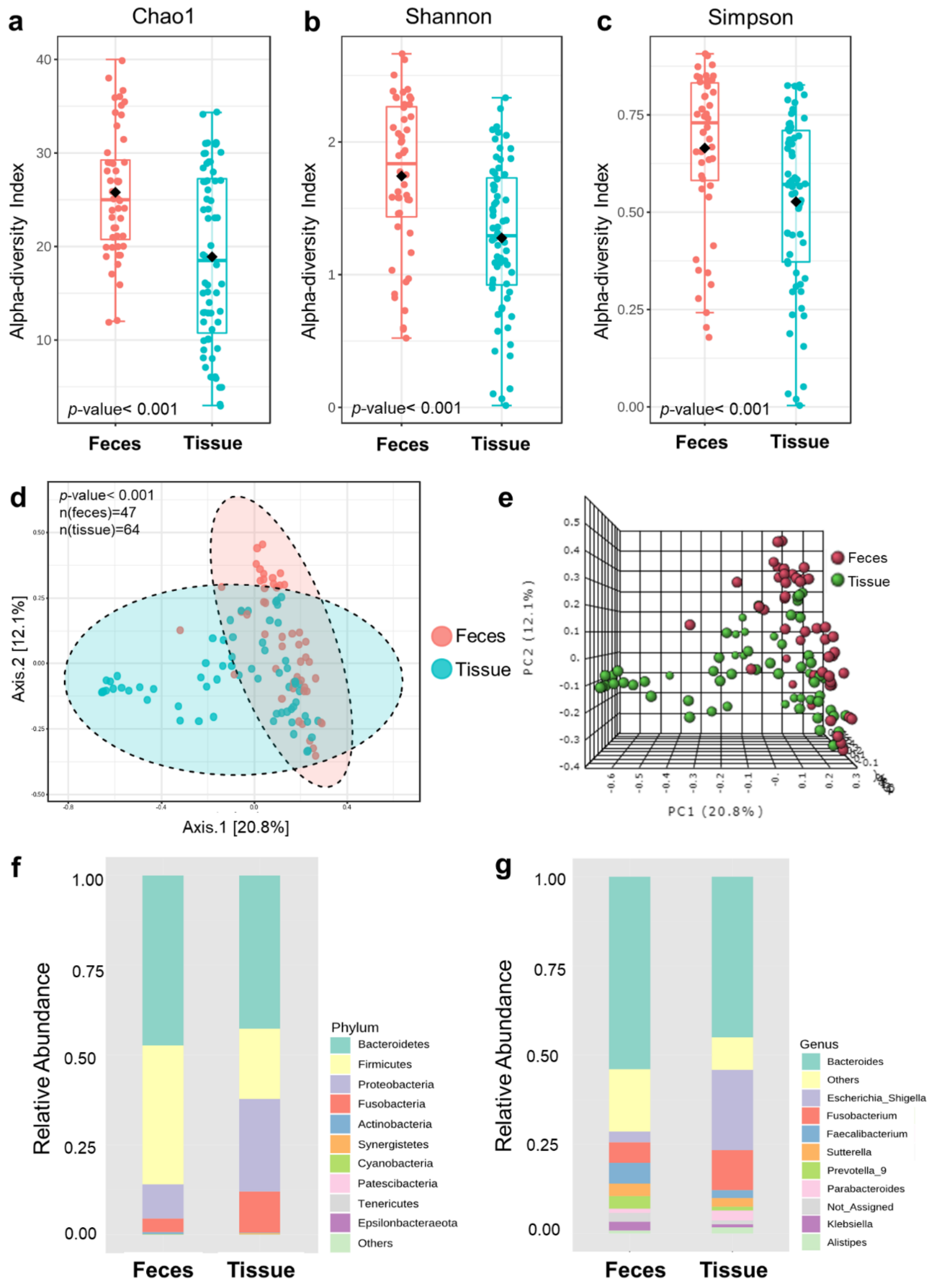

Supplementary Figure S1. Microbial diversity analyses and microbial abundance profiling between paired feces and mucosal tissue samples (a) Chao1's index, (b) Shannon's index, (c) Simpson's index, (d) Principal Coordinate Analysis, (PCoA) analysis based on Bray-Curtis distance in 2-Dimension, (e) PCoA analysis based on Bray-Curtis

distance in 3-Dimension, (f) Taxonomic composition type at the phylum level, (g) Taxonomic composition at the genus level.

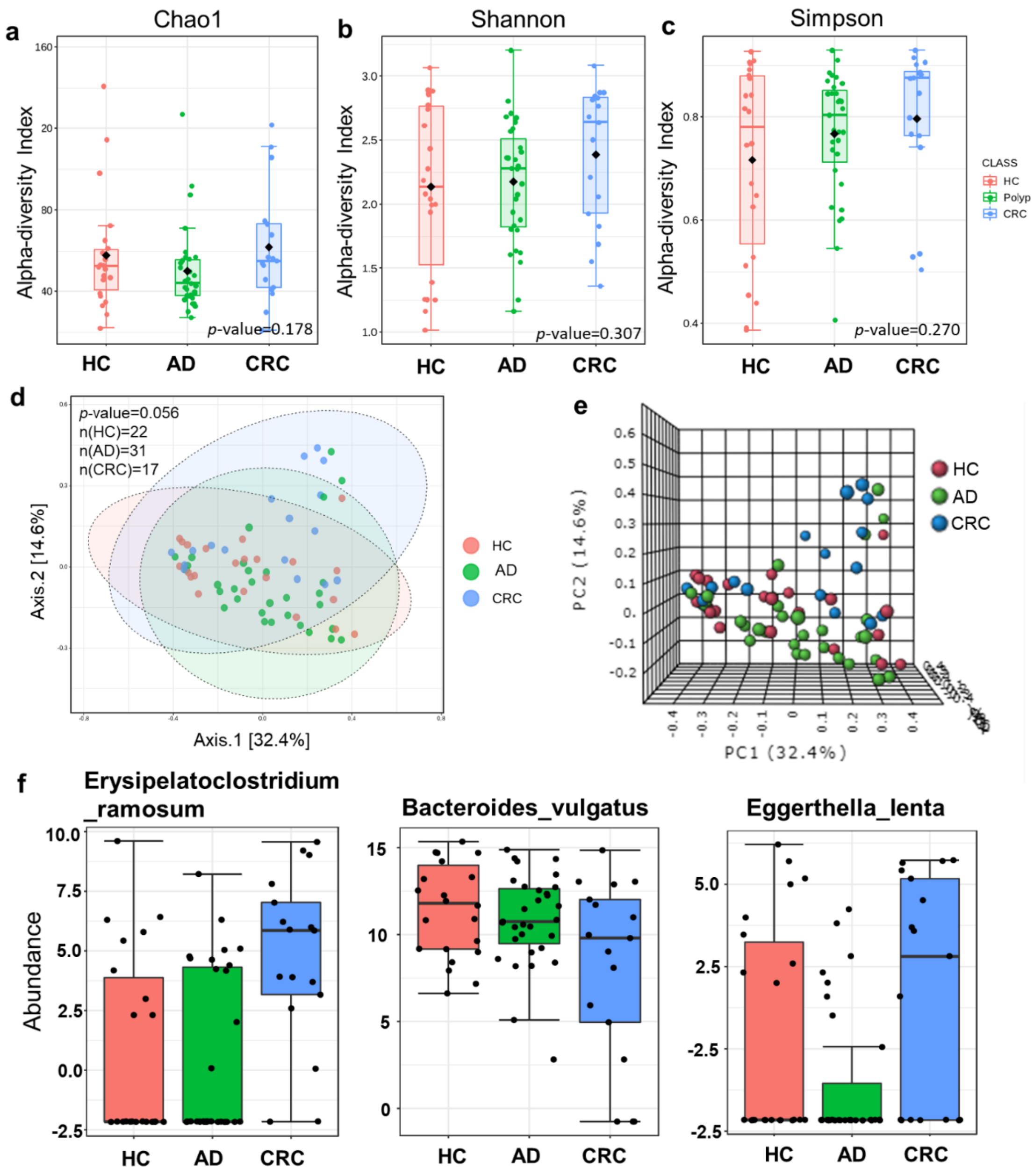

Supplementary Figure S2. Microbial diversity analyses and the significant abundance of individual bacterium at the species level in fecal samples (a) Chao1's index, (b) Shannon's index, (c) Simpson's index, (d) Principal Coordinate Analysis (PCoA) analysis based on Bray-Curtis distance in 2-Dimension, (e) PCoA analysis based on Bray-Curtis distance in 3-Dimension, **(**f) The significant difference in bacterial abundance of individual bacterium at the species level in fecal samples among healthy control (HC), adenoma (AD), colorectal cancer (CRC) subjects. Data are shown as log-transformed count and *p*-value <0.05.

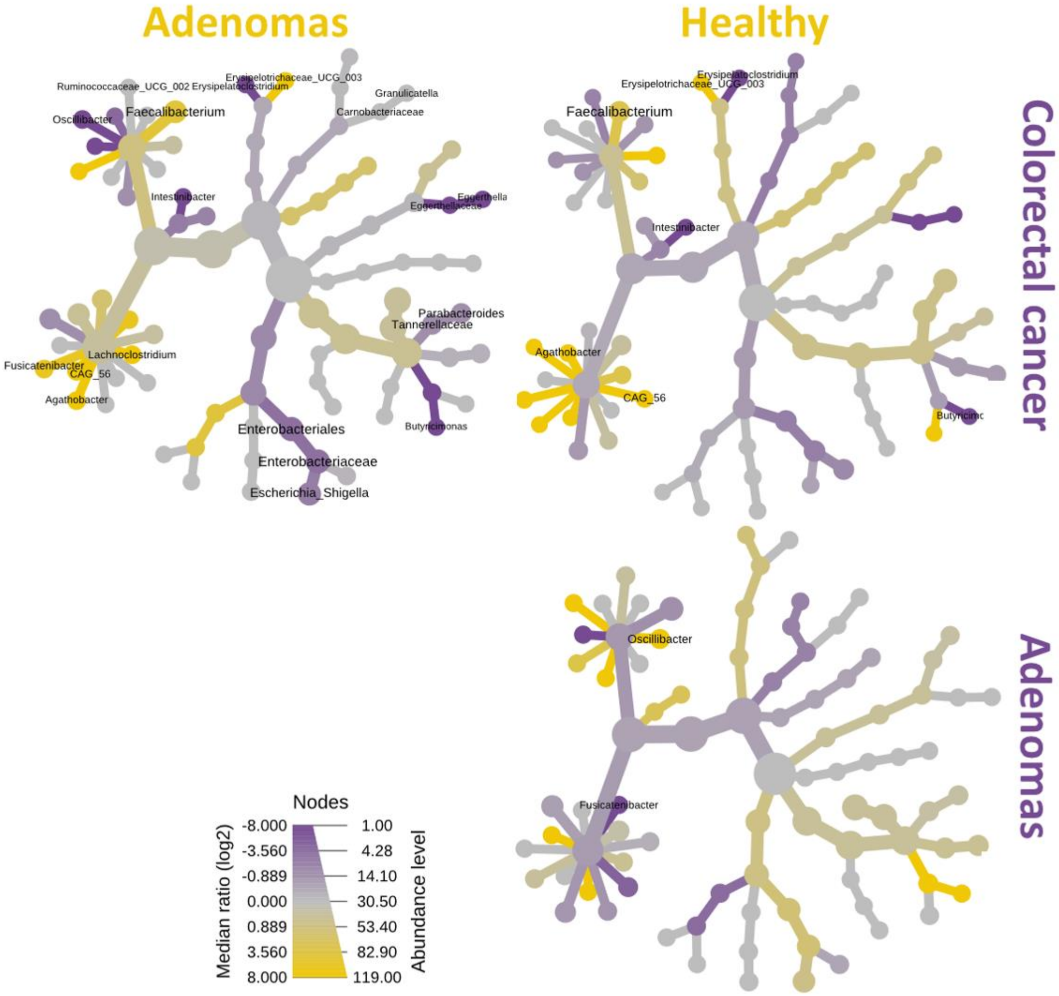

Supplementary Figure S3. Heat tree analysis illustrating the taxonomic alterations in fecal microbiota composition according to adenoma-carcinoma sequence based on the log2 ratio median abundance. Significant alterations of taxa are displayed by name at the corresponding node. The nodes indicate the hierarchical structure of taxa. Three comparisons were conducted: colorectal cancer (CRC) (purple) vs healthy control (HC) (yellow); CRC (purple) vs adenoma (AD) (yellow); and AD (purple) vs HC (yellow). The dominant color corresponds to a higher number of amplicon sequence variants (ASVs). The Log2 ratio is 0 (gray) when the compared groups are not significantly different.

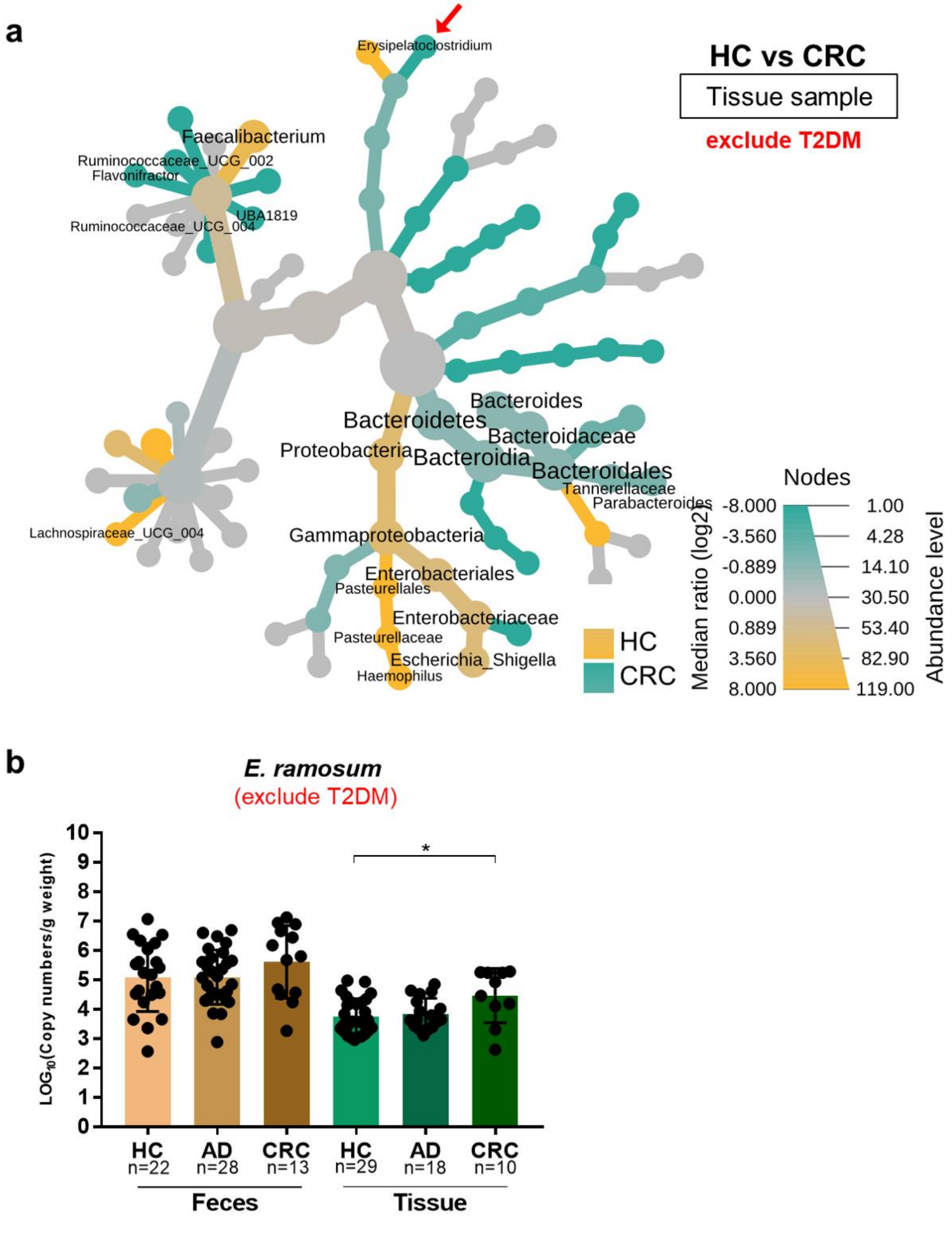

Supplementary Figure S4. The abundance of *Erysipelatoclostridium ramosum* in mucosal tissues in healthy control (HC) and colorectal cancer (CRC) subjects after exclusion of cases with type 2 diabetes mellitus (T2DM) (a) Relative abundance of *E. ramosum* by analysis of 16S rRNA gene sequencing results, (b) Absolute abundance of *E. ramosum* by quantitative PCR.
